# Effect of antibody-mediated connective tissue growth factor neutralization on lung edema in ventilator-induced lung injury in rats

**DOI:** 10.1186/s10020-024-00829-4

**Published:** 2024-05-22

**Authors:** Charissa E. van den Brom, Caitlin Bozic, Chantal A. Polet, Annabel Bongers, Anita M. Tuip-de Boer, Roselique Ibelings, Joris J. T. H. Roelofs, Nicole P. Juffermans

**Affiliations:** 1 Department of Anesthesiology, Amsterdam UMC, VU University, Amsterdam, The Netherlands; 2 Laboratory for Experimental Intensive Care and Anesthesiology (LEICA), Amsterdam UMC, University of Amsterdam, Amsterdam, The Netherlands; 3 Department of Intensive Care Medicine, Amsterdam UMC, University of Amsterdam, Amsterdam, The Netherlands; 4 Department of Pathology, Amsterdam UMC, University of Amsterdam, Amsterdam, The Netherlands; 5 Amsterdam Cardiovascular Sciences, University of Amsterdam, Amsterdam, The Netherlands; 6 OLVG Hospital, Amsterdam, The Netherlands

**Keywords:** ARDS, CTGF, Edema, Lung, Ventilator-induced lung injury

## Abstract

**Background:**

Acute respiratory distress syndrome (ARDS) is characterized by alveolar edema that can progress to septal fibrosis. Mechanical ventilation can augment lung injury, termed ventilator-induced lung injury (VILI). Connective tissue growth factor (CTGF), a mediator of fibrosis, is increased in ARDS patients. Blocking CTGF inhibits fibrosis and possibly vascular leakage. This study investigated whether neutralizing CTGF reduces pulmonary edema in VILI.

**Methods:**

Following LPS administration, rats were mechanically ventilated for 6 h with low (6 mL/kg; low V_T_) or moderate (10 mL/kg; mod V_T_) tidal volume and treated with a neutralizing CTGF antibody (FG-3154) or placebo lgG (vehicle). Control rats without LPS were ventilated for 6 h with low V_T_. Lung wet-to-dry weight ratio, FITC-labeled dextran permeability, histopathology, and soluble RAGE were determined.

**Results:**

VILI was characterized by reduced PaO_2_/FiO_2_ ratio (low V_T_: 540 [381–661] vs. control: 693 [620–754], p < 0.05), increased wet-to-dry weight ratio (low V_T_: 4.8 [4.6–4.9] vs. control: 4.5 [4.4–4.6], p < 0.05), pneumonia (low V_T_: 30 [0–58] vs. control: 0 [0–0]%, p < 0.05) and interstitial inflammation (low V_T_: 2 [1–3] vs. control: 1 [0–1], p < 0.05). FG-3154 did not affect wet-to-dry weight ratio (mod V_T_ + FG-3154: 4.8 [4.7–5.0] vs. mod V_T_ + vehicle: 4.8 [4.8–5.0], p > 0.99), extravasated dextrans (mod V_T_ + FG-3154: 0.06 [0.04–0.09] vs. mod V_T_ + vehicle: 0.04 [0.03–0.09] µg/mg tissue, p > 0.99), sRAGE (mod V_T_ + FG-3154: 1865 [1628–2252] vs. mod V_T_ + vehicle: 1885 [1695–2159] pg/mL, p > 0.99) or histopathology.

**Conclusions:**

‘Double hit’ VILI was characterized by inflammation, impaired oxygenation, pulmonary edema and histopathological lung injury. Blocking CTGF does not improve oxygenation nor reduce pulmonary edema in rats with VILI.

**Graphical Abstract:**

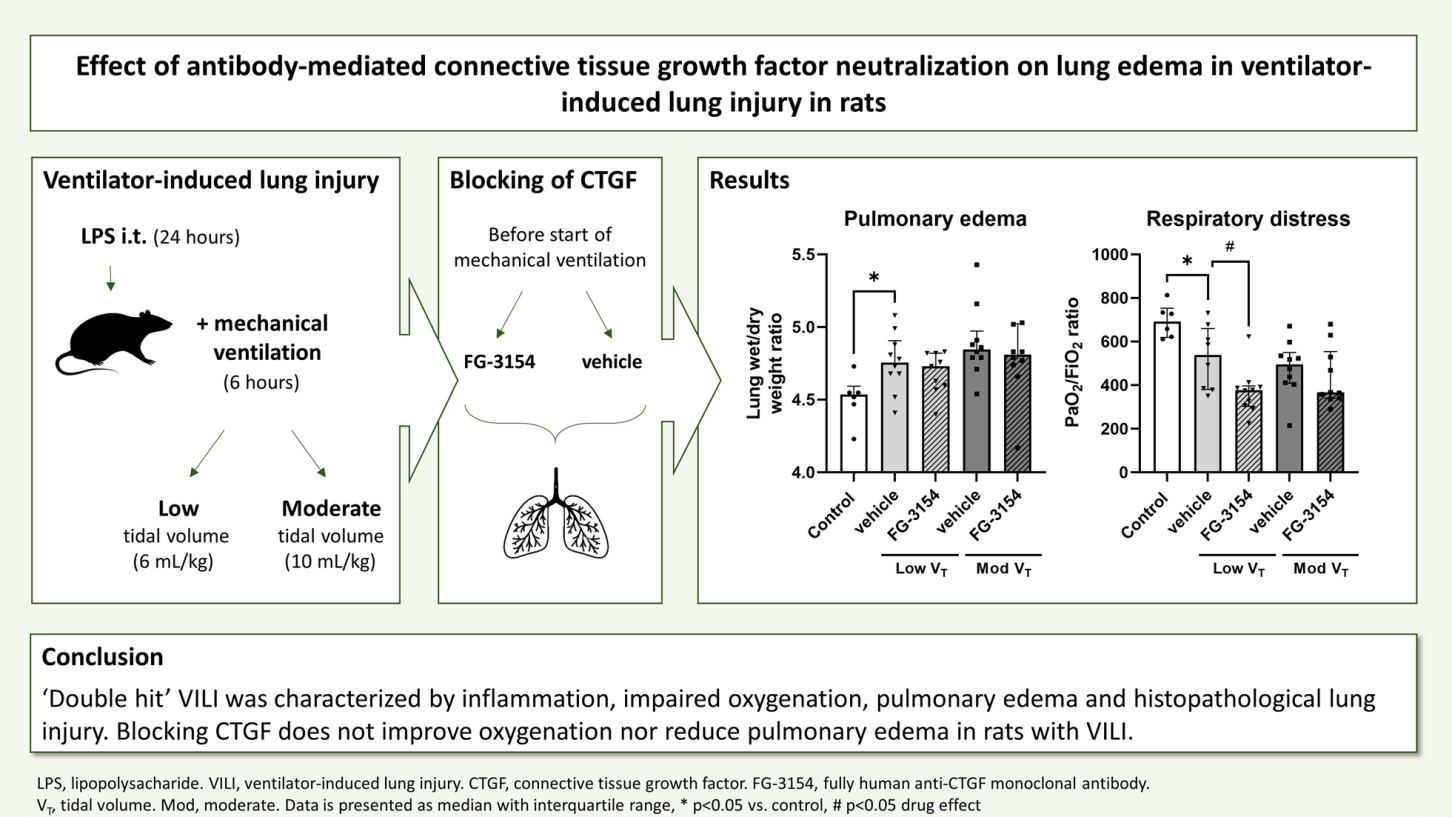

## Background

Acute respiratory distress syndrome (ARDS) complicates the course of 30% of critically ill patients admitted to the intensive care unit (ICU), and carries a strikingly high mortality of 40% (Bellani et al. 2016). Pathologic features of ARDS evolve over time. The acute phase is characterized by interstitial and alveolar edema with inflammation and hyaline membranes, collectively referred to as diffuse alveolar damage. During the course of disease, alveolar septal fibrosis can occur (Bos and Ware 2022).

Patients with ARDS often require invasive mechanical ventilation, which can amplify lung injury, referred to as ventilator-induced lung injury (VILI). Risk of VILI increases when injurious mechanical ventilation settings such as large tidal volumes or pressures are applied, and particularly when an inflammatory insult in the host is present. There is currently no specific treatment for ARDS or VILI. We hypothesized that blocking connective tissue growth factor (CTGF) may be a novel therapeutic angle.

CTGF is a matricellular protein that regulates tissue repair and fibrosis. CTGF plays a role in the pathophysiology of ARDS by promoting extracellular matrix production, leading to pulmonary fibrosis and impaired gas exchange. In experimental VILI and ARDS models, pulmonary levels of CTFG are increased and related to the severity of injurious ventilation (Wallace et al. 2009; Wu et al. 2008; Aoyama et al. 2023; Silasi-Mansat et al. 2015; Sun et al. 2023). In ARDS patients that go on to develop fibrosis, circulating levels of CTGF are increased and associated with the amount of mechanical power (Xie et al. 2019), which is the sum of the stress and strain on the lungs applied by the ventilator. In various experimental models of pulmonary fibrosis, blocking of CTGF inhibits bleomycin-induced lung fibrosis (Wang et al. 2011), radiation-induced lung fibrosis (Bickelhaupt et al. 2017) and silica-induced lung fibrosis (Cui et al. 2018). In addition, in earlier studies in patients with idiopathic pulmonary fibrosis, blocking of CTGF slowed disease progression (Cui et al. 2018).

Besides influencing fibrosis, CTFG is suggested to play a role in the regulation of vascular permeability (Lipson et al. 2012). In an experimental model of lung injury, increased CTGF levels were paralleled by pulmonary edema (Yang et al. 2015). Moreover, blocking of CTGF reduced pulmonary interstitial edema in an experimental fibrosis model (Bickelhaupt et al. 2017). Probably, vascular permeability is due to disruption of epithelial barrier function during (injurious) mechanical ventilation settings (Wu et al. 2008; Frank et al. 2006; Eyal et al. 2007). In vitro cyclic stretch of alveolar epithelial cells, representing excessive stretch during injurious ventilation, indeed increased CTGF levels and paracellular permeability (Ran et al. 2023).

Together, these data suggest that CTGF may mediate aspects of ARDS and VILI and that a CTGF neutralizing antibody may attenuate lung damage. Using a ‘double hit’ VILI model by combining an inflammatory hit with mechanical ventilation, we have tested the hypothesis that blocking of CTGF would result in less pulmonary edema and improved pulmonary histopathological lung injury.

## Methods

### Experimental set-up

All procedures were approved by the Institutional Animal Care and Use Committee of the Amsterdam Medical Center, the Netherlands (Animal welfare number: AVD11800202114545; local protocol number: 21-14545-1), conducted following the EU Directive (2010/63EU) on the protection of vertebrate animals used for experimental and other scientific purposes and reported in accordance with the Animal Research: Reporting of In Vivo Experiments (ARRIVE) guidelines (Kilkenny et al. 2010).

**Fig. 1:**
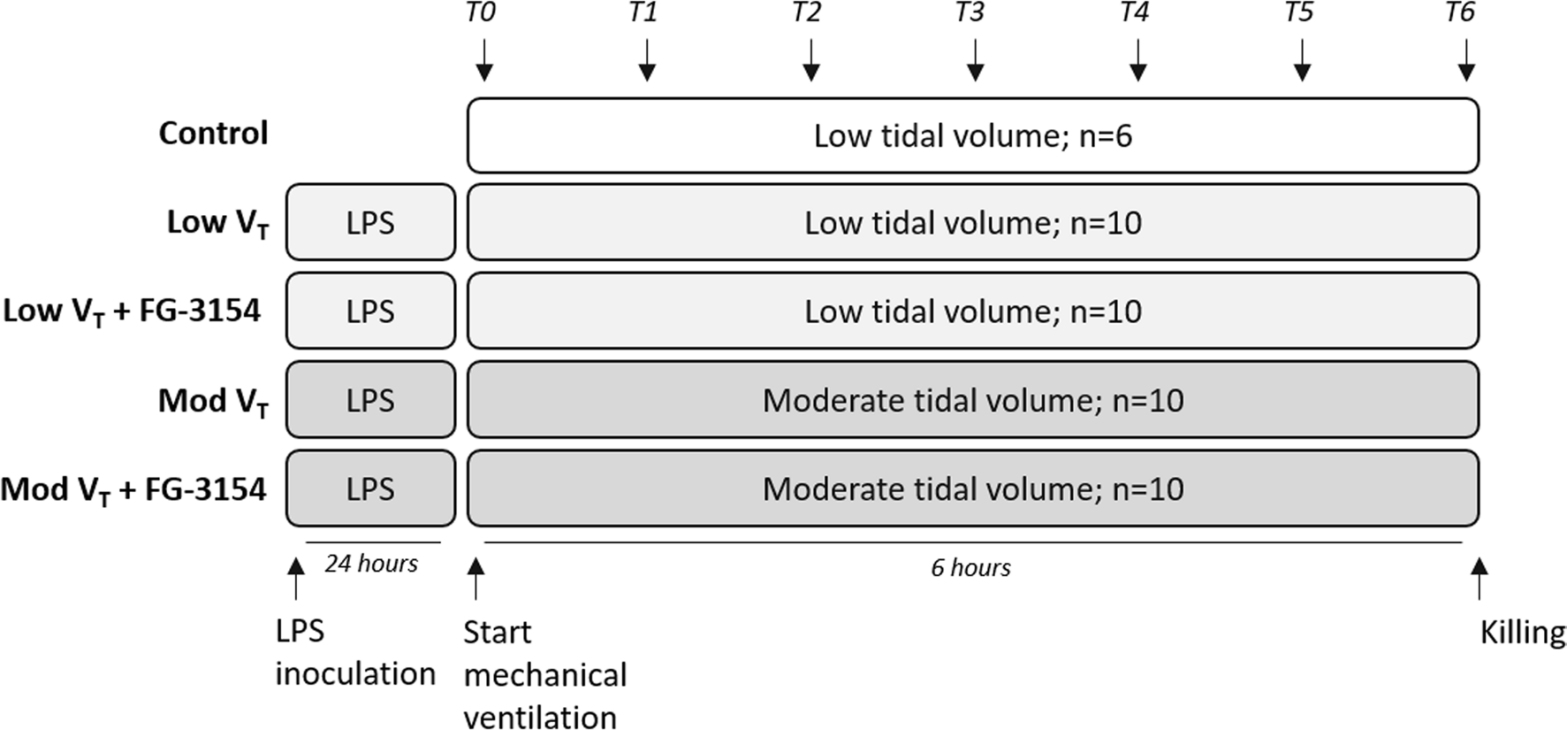
Experimental set-up. Twenty-four hours after LPS administration, rats were randomly assigned to undergo mechanical ventilation with low (6 mL/kg; Low V_T_) or moderate tidal volume (10 mL/kg; Mod V_T_) for 6 h. Immediately at start of mechanical ventilation, rats from both ventilation strategy groups received either FG-3154 treatment or vehicle as control in a blinded manner. As control, rats were mechanically ventilated with low tidal volume for 6 h without LPS administration (control). After killing, lungs were isolated and blood samples were collected and stored at − 80 °C for further analyses

Male Wistar rats weighing 345 [322–360] gram (RccHan, Envigo RMS B.V., Horst, the Netherlands) were housed three in a cage in a temperature-controlled room (12/12 h light dark cycle, 20–23 °C, 40–60% humidity) with food and water ad libitum. A well-established and frequently used two-hit lung injury model was induced by administration of lipopolysaccharides (LPS) followed by injurious mechanical ventilation (Fig. 1A). Twenty-four hours after LPS administration, rats were randomly assigned to undergo mechanical ventilation with low (low V_T_; n = 20) or moderate (mod V_T_; n = 20) tidal volume for 6 h. Immediately at start of mechanical ventilation, rats from both ventilation strategy groups received either FG-3154 treatment (n = 10) or vehicle as control (n = 10) in a blinded manner. As control, 6 rats were mechanically ventilated with low tidal volume for 6 h without LPS administration (control) and were left untreated. After killing, lungs were isolated and blood samples were collected and stored at − 80 °C for further analyses.

### LPS administration

Twenty-four hours prior to mechanical ventilation, mild lung injury was induced by LPS administration. After sedation of the rats with 4% isoflurane, lungs were intubated with a 16G catheter (Venflon Pro, Becton Dickinson, Helsingborg, Sweden) and LPS of *Escherichia Coli* (7.5 mg/kg, Sigma-Aldrich, Saint Louis, Missouri, USA) was inoculated intratracheally in a total volume of 100 µl sterile saline. Subsequently, the tracheal tube was rinsed twice with 150 µl of air and removed. Rats were intensively observed for 24 h.

### Anesthesia, surgical preparation and monitoring

Twenty-four hours after LPS administration, rats were sedated with a mixture of ketamine (90 mg/kg; Alfasan, Woerden, the Netherlands), dexmedetomidine (0.125 mg/kg; Pfizer Animal Health B.V., Capelle a/d IJssel, the Netherlands) and atropine (50 µg/kg; Pharmachemie, Haarlem, the Netherlands) intraperitoneally. A 22G catheter (Venflon Pro, Becton Dickinson, Helsingborg, Sweden) was placed in the tail vein for maintenance anesthesia which consisted of ketamine (50 mg/kg/h) and dexmedetomidine (15 µg/kg/h). Lactated Ringer (Baxter BV, Utrecht, the Netherlands) was administered as maintenance fluid at a rate of 2.5 ml/kg/h.

A 22G catheter (Venflon Pro, Becton Dickinson, Helsingborg, Sweden) was placed in the carotid artery for continuous measurements of arterial blood pressure and to allow for blood sampling for blood gas analysis (RAPIDPoint 500, Siemens, Munich, Germany). Arterial blood pressure and heart rate were continuously recorded using PowerLab software (PowerLab 8/35, Chart 8.1.2; AD Instruments Pty, Ltd., Castle Hill, Australia). Rectal temperature was maintained between 36.5 and 37.5 °C using a heating path.

### FG-3154 treatment

Before start of mechanical ventilation, rats received FG-3154, a fully human anti-CTGF monoclonal antibody (20 mg/kg; FG-3154; FibroGen Inc., San Francisco, CA, USA) or a human control lgG (vehicle; FibroGen Inc., San Francisco, CA, USA) dissolved in histidine buffered saline in a blinded manner via the tail vein.

### Mechanical ventilation

After a tracheotomy was performed, three rats were simultaneously mechanically ventilated (Babylog© 8000, Dräger, Germany) for six hours with either a low tidal volume (6 mL/kg; low V_T_) or a moderate tidal volume (10 mL/kg; mod V_T_) monitored through a differential pressure transducer (Pneumotach, HSE, Holliston). Inspired oxygen fraction (FiO_2_) was started at 0.28 and inspiratory/expiratory (I/E) ratio was kept at 1/1.5 throughout the experiment.

Low V_T_ rats were ventilated with a positive end-expiratory pressure (PEEP) of 3 cmH_2_O and a peak inspiratory pressure (PIP) of 15 cmH_2_O. Moderate V_T_ rats were ventilated with a PEEP of 5 cmH_2_O and a peak PIP to 20 cmH_2_O. Respiratory rate was adjusted based on blood gas values to maintain pH and partial pressure of carbon dioxide within physiological limits. When bicarbonate levels dropped below 18 mmoL/L, 8.4% sodium bicarbonate (Nederland B.V., Huis ter Heide, The Netherlands) was administered. Every 30 min a lung recruitment maneuver was performed by increasing PIP to 20 cmH_2_O for 3 s. When ventilator asynchrony was noticed, 0.1 mL rocuronium bromide (10 mg/mL; Organon, Oss, The Netherlands) was administered. P/F ratio was calculated by dividing the arterial PaO_2_ from the last arterial blood gas analysis by the FiO_2_.

Six hours after start of mechanical ventilation, rats were killed by extracting blood from the carotid artery and collected in EDTA tubes. The left lung was tied off and the organs were flushed with 0.9% NaCl. The left unflushed lung was used to determine wet-to-dry weight ratio. The upper lobe of the right lung was stored in formalin for histological assessment. The middle and lower lobe of the right lung was snap frozen in liquid nitrogen and stored in a − 80 °C for FITC-labeled dextran analysis.

### Pulmonary edema

The left unflushed lung was harvested at the end of the experiment. Wet tissue was weighed and dried at 37 °C. After 1 week, dry tissue was weighed and wet-to-dry weight ratio was calculated as estimate for tissue water content.

### Vascular leakage

Vascular leakage was determined by extravasation of FITC-labeled dextrans. Fifteen minutes before killing the rats, 0.5 mL FITC-labeled dextrans of 70 kDa (12.5 mg/mL; FD70S-1G, Sigma-Aldrich; Saint Louis, Missouri, USA) were administered intravenously. Fifty mg lung tissue was homogenized in RIPA buffer with protease and phosphatase inhibitors (cOmplete™ Protein Inhibitor Cocktail, Roche) and centrifuged for 15 min at 13,000 rpm at 4 °C. Fluorescence intensity of lung homogenates was determined at an excitation wavelength of 485 nm and an emission wavelength of 528 nm using a spectrophotometer (SpectraMax® M2e) and converted to µg/mg tissue using a standard curve.

### Lung histopathology

Lungs were fixed in 4% formalin and embedded in paraffin. Four-micrometer-thick paraffin sections were stained with hematoxylin and eosin. Pulmonary sections were scored by a pathologist who was blinded for treatment allocation for the following items: hemorrhage; interstitial inflammation; endothelialitis; bronchitis; perivascular edema, alveolar edema and fibrosis according a modified VILI histology scoring system as previously described (Nishina et al. 1998; Belperio et al. 2002). A score of 0 represented normal lungs; 1 represented mild, less than 25% lung involvement; 2 represented moderate, 25 to 50% lung involvement; 3 represented severe, 50 to 75% lung involvement; and 4 represented very severe, more than 75% lung involvement. Additionally, the % of the lung surface with pneumonia was scored.

### Plasma analyses

Arterial blood was collected in EDTA tubes and centrifuged at 2000 rcf for 15 min at 4 ℃. Plasma levels of soluble Receptor for Advanced Glycation End product (sRAGE, MBS7606870, MyBioSource) as marker of epithelial injury was measured with ELISA in accordance to the manufacturer.

### Statistical analysis

Data was de-blinded by the principle investigator when all data had been obtained. The normality of data distribution was determined using the Shapiro–Wilk test. As data appeared non-normally distributed, all data are expressed as median with interquartile range and analyzed using GraphPad Prism 9.0 (GraphPad Software, La Jolla, CA, USA). Primary outcome was lung wet-to-dry weight ratio. Sample size was calculated based on an estimated expected difference of 0.35 in lung wet-to-dry weight ratio in the disease model (LPS with moderate V_T_) between treatment and vehicle. The estimated effect in rats with LPS and low V_T_ was 0.15. A common standard deviation of 0.25 was used. A two-way analysis of variance with alpha of 0.05 and power of 80% revealed n = 10 per group to detect a difference between the intervention groups. In the healthy controls, n = 6 was chosen due to less variation in the group. Animals that dropped out before the end of the experiment were replaced. Animals that reached the end of the experiment were included in the analysis. The effect of LPS with low V_T_ mechanical ventilation compared to control rats was evaluated by a Mann–Whitney test. The effect of neutralizing CTGF was evaluated by Kruskal–Wallis testing with Dunn’s post hoc analyses. P values < 0.05 were considered as statistically significant.

## Results

### Hemodynamic and ventilatory monitoring

**Fig. 2:**
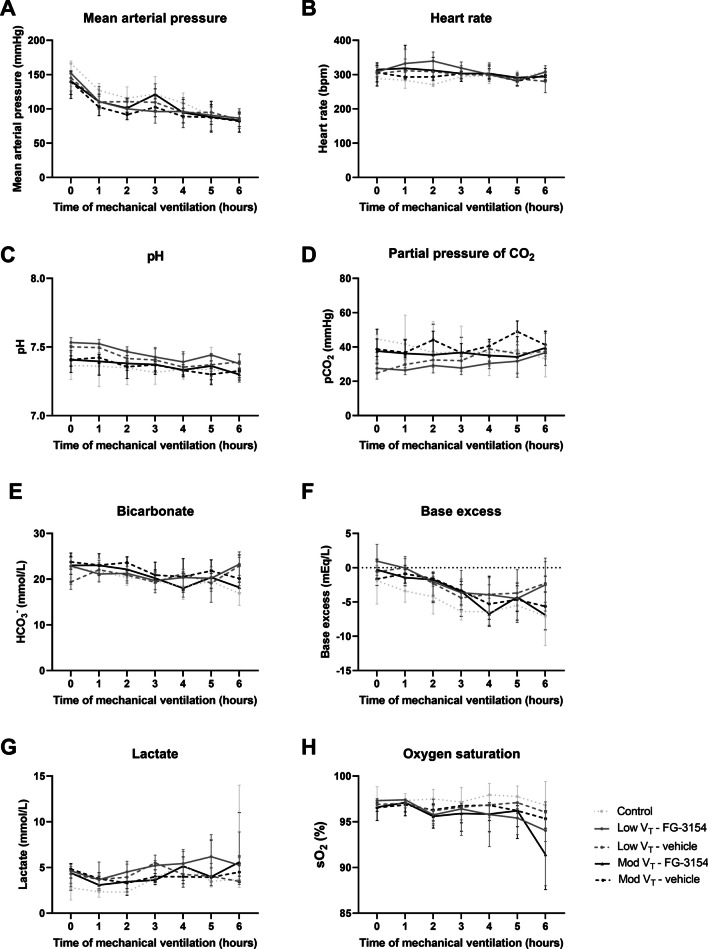
Hemodynamic parameters and blood gas analysis. Mean arterial pressure (**A**), heart rate (**B**), pH (**C**), partial pressure of CO_2_ (pCO_2_; **D**), bicarbonate (HCO_3_^−^; **E**), base excess (**F**), lactate levels (**G**) and oxygen saturation (sO_2_; **H**) in rats following LPS administration and mechanical ventilation with low (low V_T_; n = 20) or moderate (mod V_T_; n = 20) tidal volume treated with a CTGF neutralizing antibody (FG-3154; n = 10) or control IgG (vehicle; n = 10). Data are presented as median with interquartile range

Body weight was lower in rats following LPS administration compared to control rats (Table 1). Mean arterial pressure slowly decreased over time during 6 h of mechanical ventilation, whereas heart rate and body temperature remained stable over time (Fig. 2A-B, Table 1). All three parameters did not differ between intervention groups.

**Table 1:** Characteristics and arterial blood gas analysis

		Low V_T_		Moderate V_T_	
	Control (n = 6)	Vehicle (n = 10)	FG-3154 (n = 10)	Vehicle (n = 10)	FG-3154 (n = 10)
Body weight (g)	344 [328–358]	315 [293–336]*	304 [294–327]	324 [310–330]	306 [287–334]
Weight loss (%)	–	8.1 [7.3–8.6]	9.7 [8.2–10.8]	7.6 [5.6–9.1]	8.4 [8.0–9.4]
Temperature (°C)	37.3 [37.0–37.6]	36.8 [36.5–37.9]	37.5 [36.8–38.0]	37.4 [37.0–37.8]	37.3 [36.6–37.7]
*Blood gas analysis at termination*					
pO_2_ (mmHg)	194 [174–211]	160 [109–206]	109 [88–124]	145 [119–159]	103 [97–169]
Ht (%)	44 [40–46]	48 [47–52]*	49 [48–55]	47 [45–48]	46 [44–48]
Hb (mmol/L)	9.3 [8.4–9.5]	10.1 [9.9–11.1]**	10.3 [10.1–11.6]	10.0 [9.5–10.2]	9.6 [9.2–10.1]
Ca^2+^ (mmol/L)	1.00 [0.67–1.27]	1.24 [1.08–1.31]	1.22 [1.15–1.26]	1.25 [1.18–1.33]	1.18 [0.63–1.27]
*Ventilatory settings at termination*					
Tidal volume (mL/kg)	5.9 [5.9–6.1]	5.9 [5.6–6.0]	6.1 [6.0–6.1]	10.1 [9.9–10.2]	10.2 [10.1–10.8]
RR (breaths/min)	54 [54–60]	60 [53–68]	57 [52–60]	30 [30–32]	30 [30–33]
*Extra administration*					
Rocuronium (mg)	1.5 [0.8–2.0]	0 [0–1.0]	0.5 [0–1.3]	1 [0–3.5]	0.5 [0–2.3]
Bicarbonate (mg)	0.75 [0–2.00]	0.75 [0–1.35]	0.75 [0–1.25]	0 [0–1.00]	0.50 [0–0.63]

*pO*_*2*_ partial pressure of oxygen, *Ht* hematocrit, *Hb* hemoglobin, *RR* respiratory rate. Data are presented as median [IQR]. * p < 0.05, ** < 0.01 vs. control rats

Arterial blood gases showed stable pCO_2_, HCO_3_^−^, pO_2_, O_2_ saturation, lactate, Ca^2+^ and glucose, whereas pH and base excess decreased over time (Fig. 2, Table 1). Groups received similar volumes of sodium bicarbonate (Table 1). Blocking CTGF had no effect on gas exchange (Fig. 2, Table 1). Hematocrit and hemoglobin did not differ over time nor between treatment groups (Table 1).

According to protocol, tidal volume was higher in rats on moderate V_T_ ventilation compared to rats on low V_T_ ventilation, without differences between treatment groups (Table 1). As a consequence, respiratory rates needed to be adjusted and were lower in moderate V_T_ rats compared to low V_T_ rats, without differences in administered volumes of rocuronium bromide (Table 1).

### Pulmonary permeability

**Fig. 3:**
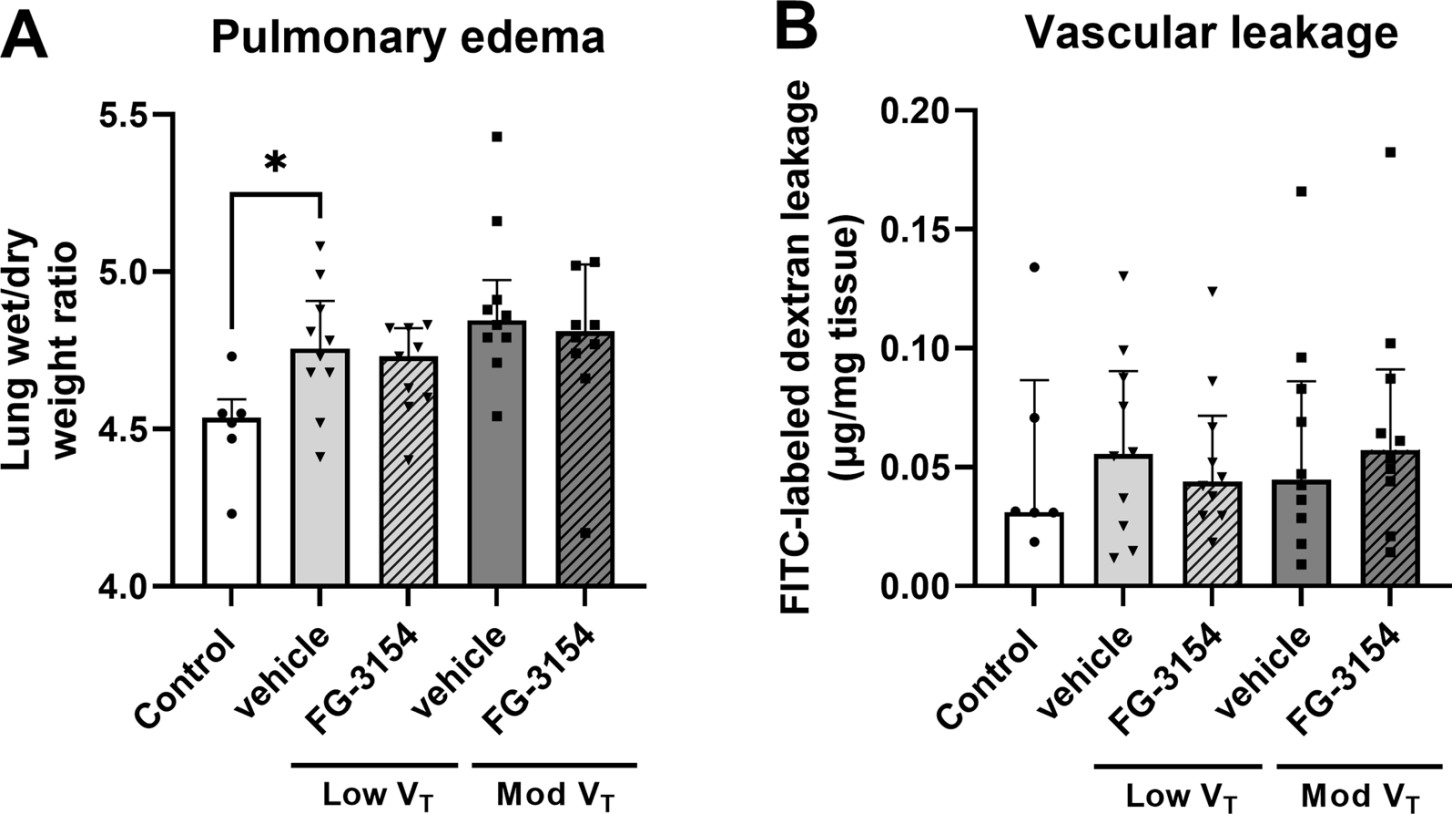
Pulmonary permeability. Pulmonary wet-to-dry weight ratio (**A**) and pulmonary FITC-labeled dextran leakage (**B**) in rats following LPS administration and 6 h of mechanical ventilation with low (low V_T_) or moderate (mod V_T_) tidal volume treated with a CTGF neutralizing antibody (FG-3154) or control IgG (vehicle). Data are presented as median with interquartile range. * p < 0.05 vs. control rats

The combination of LPS and mechanical ventilation increased pulmonary wet-to-dry weight ratio (Fig. 3A), but not accumulation of FITC-labeled dextrans in lung tissue (Fig. 3B) compared to control rats. Blocking CTGF did not affect pulmonary wet-to-dry weight ratio nor accumulation of FITC-labeled dextrans (Fig. 3).

### Lung injury

**Fig. 4:**
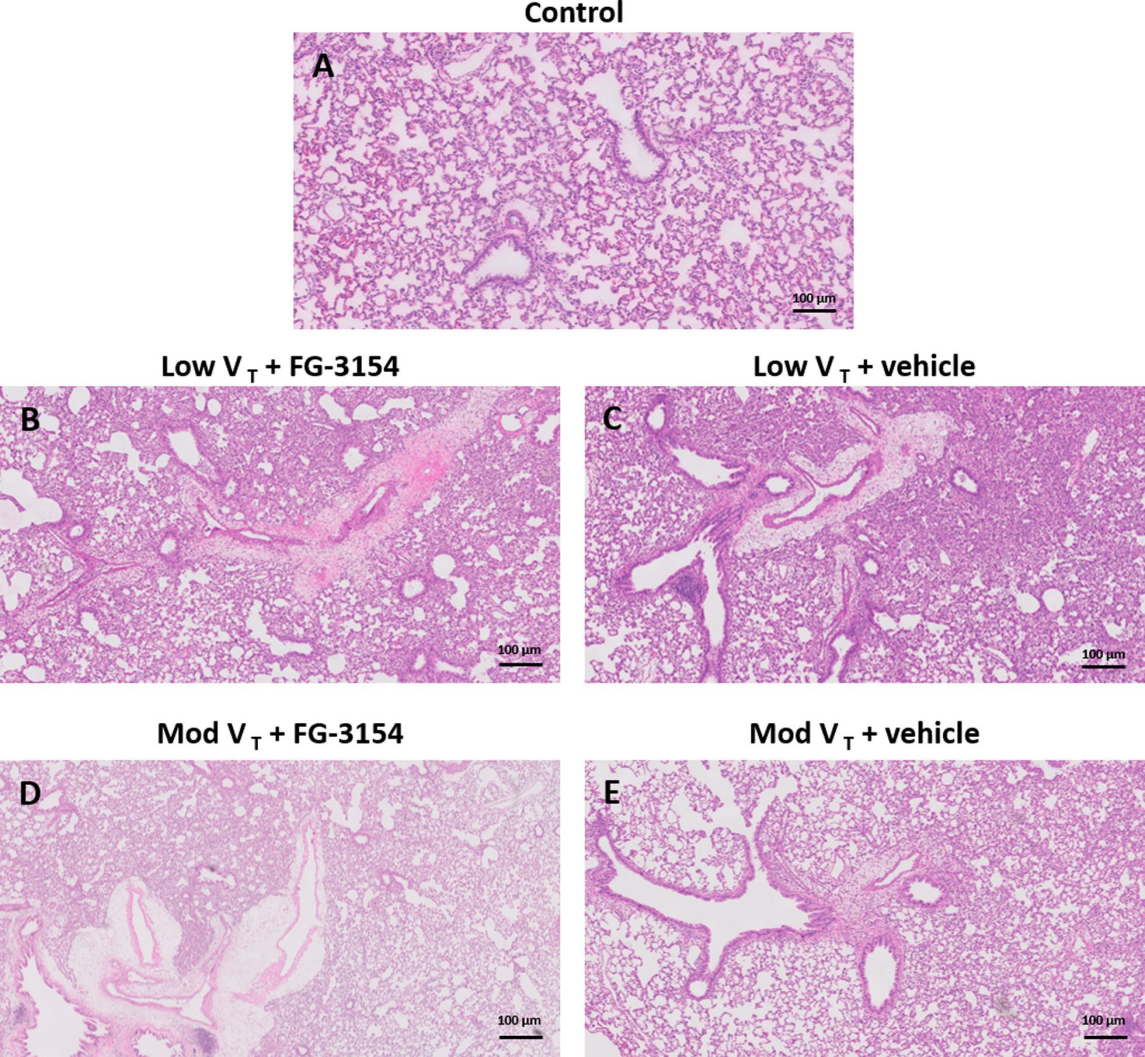
Histopathology. Microscopy of representative hematoxylin and eosin stained sections of rat lung tissue. Tissue from control rats (**A**), tissue from rats following LPS administration and 6 h of mechanical ventilation with low (low V_T_) tidal volume treated with CTGF neutralizing antibody (FG-3154) (**B**) or control IgG (vehicle) (**C**) and tissue from rats following LPS administration and 6 h of mechanical ventilation with moderate (mod V_T_) tidal volume treated with CTGF neutralizing antibody (FG-3154; n = 10) (**D**) or control IgG (vehicle) (**E**)

**Fig. 5:**
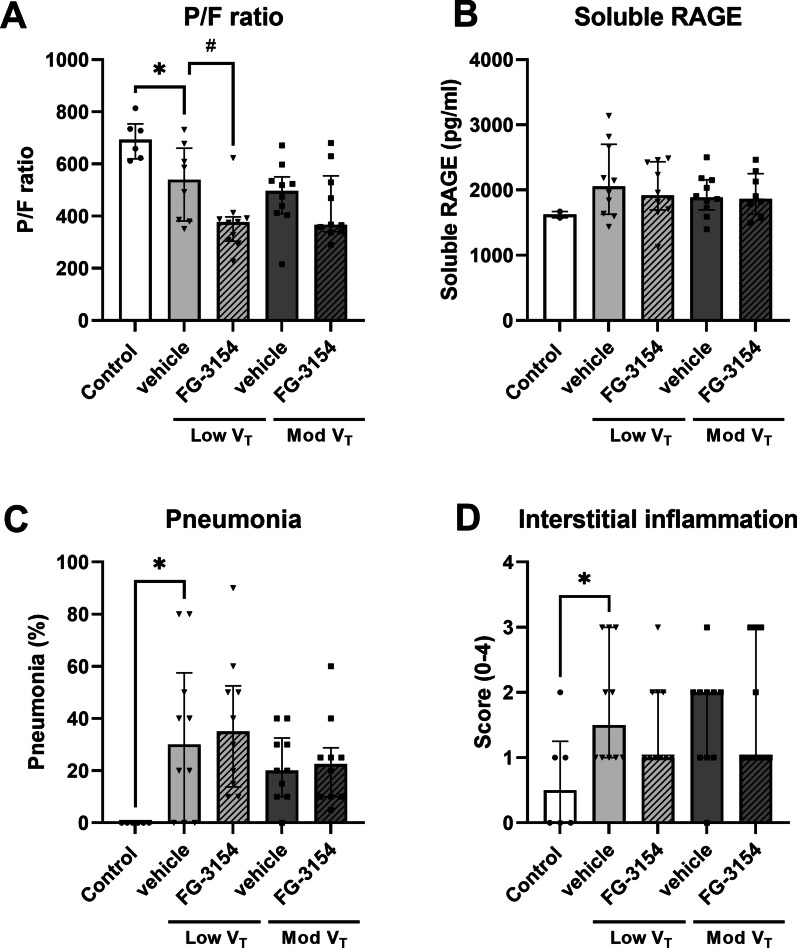
Lung injury. PaO_2_/FiO_2_ ratio (P/F ratio; **A**), soluble Receptor for Advanced Glycation End product (sRAGE; **B**), histological assessment of pneumonia (**C**) and histological assessment of interstitial inflammation (**D**) in rats following LPS administration and 6 h of mechanical ventilation with low (low V_T_) or moderate (mod V_T_) tidal volume treated with CTGF neutralizing antibody (FG-3154) or control IgG (vehicle). Data are presented as median with interquartile range. * p < 0.05 vs. control rats, # p < 0.05 vs. vehicle

LPS administration and mechanical ventilation reduced P/F ratio compared to controls (Fig. 5A). Histopathological assessment showed increased pneumonia (Figs. 4 and 5C) and interstitial inflammation (Figs. 4 and 5D) in rats following LPS and mechanical ventilation compared to controls, but no signs of hemorrhage, endothelialitis, bronchitis, perivascular, alveolar edema or fibrosis were present (Table 2).

**Table 2:** Lung histopathology

		Low V_T_		Moderate V_T_	
	Control (n = 6)	Vehicle (n = 10)	FG-3154 (n = 10)	Vehicle (n = 10)	FG-3154 (n = 10)
Hemorrhage	0 [0–0]	0 [0.0–0.3]	0 [0–0]	0 [0–0]	0 [0–0]
Endothelialitits	0 [0–0]	0 [0–0]	0 [0–0]	0 [0–0]	0 [0–0]
Bronchitis	0 [0.0–0.3]	1 [0.0–2.0]	1 [0.0–2.0]	1.5 [0–2]	1 [0–2]
Perivascular edema	2 [2–2]	2 [2–3]	2 [2–2]	2 [2–3]	3 [2–3]
Alveolar edema	0 [0–0]	0 [0–0]	0 [0–1]	0 [0–0]	0 [0–0]
Fibrosis	0 [0–0]	0 [0–0]	0 [0–0]	0 [0–0]	0 [0–0]

Data are presented as median [IQR] based on a score of 0 to 4. A score of 0 represented normal lungs; 1 represented mild, less than 25% lung involvement; 2 represented moderate, 25 to 50% lung involvement; 3 represented severe, 50 to 75% lung involvement; and 4 represented very severe, more than 75% lung involvement

Blocking CTFG did not improve P/F ratio and circulating levels of soluble RAGE (Fig. 5A-B) nor histopathological signs of lung injury (Fig. 4C-D, Table 2).

## Discussion

Connective tissue growth factor (CTGF) is a mediator of fibrosis, but CTGF is also suggested to be involved in the regulation of vascular permeability. In the present study, we have investigated whether neutralizing CTGF reduced pulmonary edema in a ‘double hit’ VILI rat model. VILI was characterized by inflammation, impaired oxygenation, pulmonary edema and histopathological lung injury. Contrary to our hypothesis, blocking of CTGF by FG-3154 had no effect on pulmonary vascular leakage and pulmonary edema formation. Blocking of CTGF also did not affect epithelial injury nor histopathological lung injury. These data showed that neutralizing CTGF by FG-3154 does not prevent the development of pulmonary edema and lung injury in a ‘double hit’ VILI rat model.

In our study, we have specifically focused on the early phase of ARDS characterized by alveolar edema. We have chosen this early model given that upregulation of CTGF occurs very early in response to injurious mechanical ventilation (Wallace et al. 2009; Wu et al. 2008; Aoyama et al. 2023). An explanation for an absence of effect may be that CTFG protein levels were not sufficiently increased in our model, which may be due to local suppressive effects of LPS on CTGF (Zhou et al. 2021). Unfortunately, we cannot provide lung CTGF protein levels in our model. However, while our study does not directly measure CTGF levels, we showed that the combination of LPS with mechanical ventilation induced inflammation, impaired oxygenation, pulmonary edema and histopathological lung injury, and that blocking of CTGF is not effective in this experimental VILI model.

Another possible explanation why there was no benefit of blocking CTGF is that we have specifically focused on alveolar edema, as it was previously suggested that inhibition of CTFG might also decrease vascular permeability. CTGF modulates inflammatory responses, which are not only precursors to the fibrotic process, but also play a role in the regulation of vascular permeability. Indeed, increased CTGF levels were paralleled by increased paracellular permeability in cultured epithelial cells exposed to cyclic stretch (Ran et al. 2023) or following thrombin administration (Akter et al. 2022). In an experimental animal model of lung injury, increased CTGF levels were paralleled by pulmonary edema (Yang et al. 2015) and blocking of CTGF reduced pulmonary interstitial edema in an experimental fibrosis model (Bickelhaupt et al. 2017). However, this is indirect evidence that CTGF is involved in regulating vascular permeability. Contrasting findings were found in in vitro settings, in which increased CTGF expression improved barrier function in retinal cells (Suzuki et al. 2022). Moreover, CTGF delivery reduced pulmonary vascular leakage and edema formation in rats with cecal ligation and puncture-induced sepsis and LPS-stimulated endothelial cells (Zhou et al. 2021). Interestingly, low dose CTGF treatment in LPS-induced acute lung injury in mice alleviated alveolar damage, whereas high dosing of CTGF resulted in pulmonary fibrosis (Sun et al. 2023), suggesting dose-dependent effects of CTGF. Based on the current literature, we speculate that CTGF might has cell-type specific effects as it seems to mostly affect alveolar epithelial cells, whereas endothelial cells also play a crucial role in maintaining vascular permeability. Taken together, the relationship between pulmonary CTGF expression and vascular permeability is complex and seems to be context and cell type-dependent. Our results suggest that neutralizing CTGF by FG-3154 does not protect against vascular hyperpermeability in an acute model of lung injury.

On the other hand, our findings are not surprising as CTGF is particularly known in the context of lung fibrosis. Promising effects of blocking CTGF by pamrevlumab has been shown in rats and in earlier studies in patients with lung fibrosis (Wang et al. 2011; Bickelhaupt et al. 2017; Cui et al. 2018; Richeldi et al. 2020). Together with the findings of the present study and a recent study in which pamrevlumab had no beneficial effect in patients with COVID19 pneumonia (Sgalla et al. 2023) this underscores the importance for selection of patients in a later stage of ARDS.

This study was associated with some limitations. We do not show additional lung injury following mechanical ventilation with a tidal volume of 10 mL/kg compared to a tidal volume of 6 mL/kg. This is possibly explained by the adjusted respiratory rate to maintain comparable acid base balances between groups, which results in a comparable mechanical power (Moraes et al. 2018). We however have shown that our rats developed inflammation, impaired oxygenation, pulmonary edema and histopathological lung injury, which is in accordance with the pathology of VILI. Moreover, considering the suggested dose-dependent effects of CTGF (Sun et al. 2023), a limitation of our study is the investigation of only a single dose of FG-3154.

Our results have potential implications in terms of designing clinical studies investigating neutralizing CTGF in ARDS. As early intervention seems not effective in reducing pulmonary edema, our results underline the rationale of selection of patients in later stages of ARDS for this treatment.

## Conclusions

The present study showed that neutralizing CTGF by FG-3154 does not prevent the development of pulmonary edema and histopathological lung injury in a ‘double hit’ VILI rat model and suggests that blocking of CTGF does not protects against vascular hyperpermeability in an acute model of lung injury. Future research should focus on whether CTGF plays a prominent role in regulation of vascular permeability, but also to identify more promising targets to protect the endothelium in order to reduce or prevent acute lung injury.

## Data Availability

The datasets used and/or analyzed during the current study are available from the corresponding author on reasonable request.

## Abbreviations

ARDS: Acute respiratory distress syndrome
ARRIVE: Animal Research Reporting of In Vivo Experiments
Ca^2+^: Calcium
CTGF: Connective tissue growth factor
EDTA: Ethylenediaminetetraacetic acid
ELISA: Enzyme-Linked Immuno Sorbent Assay
FiO2: Inspired oxygen fraction
FITC: Fluorescein isothiocyanate
HCO_3_^−^: Bicarbonate
ICU: Intensive care unit
I/E: Inspiratory/expiratory ratio
LPS: Lipopolysaccharides
O_2_: Oxygen
pCO_2_: Partial pressure of carbon dioxide
PEEP: Positive end-expiratory pressure
P/F: PO_2_/FiO_2_
PIP: Peak inspiratory pressure
pO_2_: Partial pressure of oxygen
RIPA: Radioimmunoprecipitation assay
sRAGE: Soluble Receptor for Advanced Glycation End product
VILI: Ventilator-induced lung injury
V_T_: Tidal volume

## References

[CR1] Akter T, Annamalai B, Obert E, Simpson KN, Rohrer B (2022). Dabigatran and wet AMD, results from retinal pigment epithelial cell monolayers, the mouse model of choroidal neovascularization, and patients from the medicare data base. Front Immunol.

[CR2] Aoyama J, Saito Y, Matsuda K, Tanaka T, Kamio K, Gemma A (2023). Increased CTGF expression in alveolar epithelial cells by cyclic mechanical stretch: its mechanism and the therapeutic effect of pirfenidone. Respir Physiol Neurobiol.

[CR3] Bellani G, Laffey JG, Pham T, Fan E, Brochard L, Esteban A, et al.; LUNG SAFE Investigators; ESICM Trials Group. Epidemiology, patterns of care, and mortality for patients with acute respiratory distress syndrome in intensive care units in 50 countries. JAMA. 2016;315:788–800. 10.1001/jama.2016.0291 26903337

[CR4] Belperio JA, Keane MP, Burdick MD, Londhe V, Xue YY, Li K (2002). Critical role for CXCR2 and CXCR2 ligands during the pathogenesis of ventilator-induced lung injury. J Clin Invest.

[CR5] Bickelhaupt S, Erbel C, Timke C, Wirkner U, Dadrich M, Flechsig P, et al. Effects of CTGF Blockade on Attenuation and Reversal of Radiation-Induced Pulmonary Fibrosis. J Natl Cancer Inst. 2017;109. 10.1093/jnci/djw339 28376190

[CR6] Bos LDJ, Ware LB (2022). Acute respiratory distress syndrome: causes, pathophysiology, and phenotypes. Lancet.

[CR7] Cui X, Peng Z, Zhou Y, Zhang H, Lipson K, Liu Y (2018). Anti-CTGF antibody attenuates silica-induced lung fibrosis in rats. Eur Resp J..

[CR8] Eyal FG, Hamm CR, Parker JC (2007). Reduction in alveolar macrophages attenuates acute ventilator induced lung injury in rats. Intensive Care Med.

[CR9] Frank JA, Wray CM, McAuley DF, Schwendener R, Matthay MA (2006). Alveolar macrophages contribute to alveolar barrier dysfunction in ventilator-induced lung injury. Am J Physiol Lung Cell Mol Physiol.

[CR10] Kilkenny C, Browne WJ, Cuthill IC, Emerson M, Altman DG (2010). Improving bioscience research reporting: the ARRIVE guidelines for reporting animal research. PLoS Biol.

[CR11] Lipson KE, Wong C, Teng Y, Spong S (2012). CTGF is a central mediator of tissue remodeling and fibrosis and its inhibition can reverse the process of fibrosis. Fibrogenesis Tissue Repair.

[CR12] Moraes L, Silva PL, Thompson A, Santos CL, Santos RS, Fernandes MVS (2018). Impact of different tidal volume levels at low mechanical power on ventilator-induced lung injury in rats. Front Physiol.

[CR13] Nishina K, Mikawa K, Takao Y, Shiga M, Maekawa N, Obara H (1998). Intravenous lidocaine attenuates acute lung injury induced by hydrochloric acid aspiration in rabbits. Anesthesiology.

[CR14] Ran X, Müller S, Brunssen C, Huhle R, Scharffenberg M, Schnabel C (2023). Modulation of the hippo-YAP pathway by cyclic stretch in rat type 2 alveolar epithelial cells-a proof-of-concept study. Front Physiol.

[CR15] Richeldi L, Fernández Pérez ER, Costabel U, Albera C, Lederer DJ, Flaherty KR (2020). Pamrevlumab, an anti-connective tissue growth factor therapy, for idiopathic pulmonary fibrosis (PRAISE): a phase 2, randomised, double-blind, placebo-controlled trial. Lancet Respir Med.

[CR16] Sgalla G, Leone PM, Gualano G, Simonetti J, Comes A, Verdirosi D (2023). A randomized trial of pamrevlumab in patients with COVID-19 pneumonia. Respirology.

[CR17] Silasi-Mansat R, Zhu H, Georgescu C, Popescu N, Keshari RS, Peer G (2015). Complement inhibition decreases early fibrogenic events in the lung of septic baboons. J Cell Mol Med.

[CR18] Sun J, Zhang H, Liu D, Liu W, Du J, Wen D, Li L (2023). CTGF promotes the repair and regeneration of alveoli after acute lung injury by promoting the proliferation of subpopulation of AEC2s. Respir Res.

[CR19] Suzuki S, Furuhashi M, Tsugeno Y, Umetsu A, Ida Y, Hikage F (2022). Comparison of the drug-induced efficacies between omidenepag isopropyl, an EP2 agonist and PGF2α toward TGF-β2-modulated human trabecular meshwork (HTM) cells. J Clin Med.

[CR20] Wallace MJ, Probyn ME, Zahra VA, Crossley K, Cole TJ, Davis PG (2009). Early biomarkers and potential mediators of ventilation-induced lung injury in very preterm lambs. Respir Res.

[CR21] Wang Q, Usinger W, Nichols B, Gray J, Xu L, Seeley TW (2011). Cooperative interaction of CTGF and TGF-β in animal models of fibrotic disease. Fibrogenesis Tissue Repair.

[CR22] Wu S, Capasso L, Lessa A, Peng J, Kasisomayajula K, Rodriguez M (2008). High tidal volume ventilation activates Smad2 and upregulates expression of connective tissue growth factor in newborn rat lung. Pediatr Res.

[CR23] Xie Y, Wang Y, Liu K, Li X (2019). Correlation analysis between mechanical power, transforming growth factor-β1, and connective tissue growth factor levels in acute respiratory distress syndrome patients and their clinical significance in pulmonary structural remodeling. Medicine (baltimore).

[CR24] Yang Z, Sun Z, Liu H, Ren Y, Shao D, Zhang W (2015). Connective tissue growth factor stimulates the proliferation, migration and differentiation of lung fibroblasts during paraquat-induced pulmonary fibrosis. Mol Med Rep.

[CR25] Zhou H, Zheng D, Wang H, Wu Y, Peng X, Li Q (2021). The protective effects of pericyte-derived microvesicles on vascular endothelial functions via CTGF delivery in sepsis. Cell Commun Signal.

